# Pre-Procedural Assessment of the Femoral Access Route for Transcatheter Aortic Valve Implantation: Comparison of a Non-Contrast Time-of-Flight Magnetic Resonance Angiography Protocol with Contrast-Enhanced Dual-Source Computed Tomography Angiography

**DOI:** 10.3390/jcm12216824

**Published:** 2023-10-29

**Authors:** Johannes Brado, Philipp Breitbart, Manuel Hein, Gregor Pache, Ramona Schmitt, Jonas Hein, Matthias Apweiler, Martin Soschynski, Christopher Schlett, Fabian Bamberg, Franz-Josef Neumann, Dirk Westermann, Tobias Krauss, Philipp Ruile

**Affiliations:** 1 Department of Cardiology and Angiology, Medical Center—University of Freiburg, Faculty of Medicine, University of Freiburg, Südring 15, 79189 Bad Krozingen, Germany; 2 Radiology Hegau Bodensee, Practice for Diagnostic Radiology, Kreuzensteinstraße 7, 78224 Singen, Germany; 3 Department of Diagnostic and Interventional Radiology, Medical Center—University of Freiburg, Faculty of Medicine, University of Freiburg, 79106 Freiburg, Germany

**Keywords:** transcatheter aortic valve implantation, renal insufficiency, MR angiography, CT angiography

## Abstract

**Background:** We aimed to evaluate the feasibility of a non-contrast time-of-flight magnetic resonance angiography (TOF-MRA) protocol for the pre-procedural access route assessment of transcatheter aortic valve implantation (TAVI) in comparison with contrast-enhanced cardiac dual-source computed tomography angiography (CTA). **Methods and Results:** In total, 51 consecutive patients (mean age: 82.69 ± 5.69 years) who had undergone a pre-TAVI cardiac CTA received TOF-MRA for a pre-procedural access route assessment. The MRA image quality was rated as very good (median of 5 [IQR 4–5] on a five-point Likert scale), with only four examinations rated as non-diagnostic. The TOF-MRA systematically underestimated the minimal effective vessel diameter in comparison with CTA (for the effective vessel diameter in mm, the right common iliac artery (CIA)/external iliac artery (EIA)/common femoral artery (CFA) MRA vs. CTA was 8.04 ± 1.46 vs. 8.37 ± 1.54 (*p* < 0.0001) and the left CIA/EIA/CFA MRA vs. CTA was 8.07 ± 1.32 vs. 8.28 ± 1.34 (*p* < 0.0001)). The absolute difference between the MRA and CTA was small (for the Bland–Altman analyses in mm, the right CIA/EIA/CFA was −0.36 ± 0.77 and the left CIA/EIA/CFA was −0.25 ± 0.61). The overall correlation between the MRA and CTA measurements was very good (with a Pearson correlation coefficient of 0.87 (*p* < 0.0001) for the right CIA/EIA/CFA and a Pearson correlation coefficient of 0.9 (*p* < 0.0001) for the left CIA/EIA/CFA). The feasibility agreement between the MRA and CTA for transfemoral access was good (the right CIA/EIA/CFA agreement was 97.9% and the left CIA/EIA/CFA agreement was 95.7%, Kohen’s kappa: 0.477 (*p* = 0.001)). **Conclusions**: The TOF-MRA protocol was feasible for the assessment of the access route in an all-comer pre-TAVI population. This protocol might be a reliable technique for patients at an increased risk of contrast-induced nephropathy.

## 1. Introduction

At present, transcatheter aortic valve implantation (TAVI) is the standard of care for patients with severe aortic stenosis and increased perioperative risks [1,2,3]. Computed tomography angiography (CTA) is the gold standard imaging tool used to plan the procedure [4,5]. This diagnostic tool permits an excellent three-dimensional assessment of the aortic annulus and the access route. CTA also facilitates the identification of predictors for increased periprocedural risks. These include the extent of the left-ventricular outflow tract (LVOT) calcification and coronary ostial height as predictors of annular ruptures or coronary occlusions, respectively, or the presence and extent of aortic arch atheroma and the extent of aortic valve calcification as predictors of postprocedural strokes [6]. CTA-based TAVI planning requires the use of intravenous iodinated contrast media. The high prevalence of impaired renal functions at the baseline in a cohort of elderly, multimorbid patients had an increased risk of contrast-induced nephropathy [7]. Contrast-induced nephropathy appears to have a negative influence on the procedural outcome [8]. Recently, published data from a large registry of over 100,000 patients revealed that more than ten percent had experienced a postprocedural acute kidney injury following transcatheter aortic valve implantation. A kidney injury stage 3 or higher is associated with a seven-fold increase in one-year mortality [9].

In a previous study, we described the feasibility and accuracy of respiratory and ECG-triggered 3D non-contrast MRA for aortic annulus assessments in comparison with contrast-enhanced CTA [10]. To date, there are sparse data on non-contrast assessments of the aortofemoral access route.

Thus, the aim of this study was to evaluate the feasibility and accuracy of a non-contrast time-of-flight magnetic resonance angiography (TOF-MRA) protocol for pre-procedural femoral access route assessments of transcatheter aortic valve implantation (TAVR) in comparison with contrast-enhanced cardiac dual-source computed tomography angiography (CTA).

## 2. Materials and Methods

### 2.1. Study Population

The potential candidates for inclusion in this study were all patients with severe symptomatic aortic stenosis who had been referred for CTA prior to a TAVI evaluation between February 2014 and March 2017. Patients were prospectively enrolled if none of the exclusion criteria were present. The exclusion criteria were a rejection of the MRA examination, the inability to remain in a supine position due to severe orthopnea or a reduced general state of health, the presence of metallic foreign bodies, a permanent pacemaker or severe claustrophobia. This prospective study was approved by the institutional review board and complied with the Declaration of Helsinki.

### 2.2. CTA Data Acquisition

CTA examinations were performed using a second-generation dual-source CT scanner (Somatom Definition Flash, Siemens healthineers, Erlangen, Germany).

In total, 50 mL of a commercially available contrast medium (Imeron 400^®^, Bracco, Konstanz, Germany) was used as an iodinated contrast agent with a dual-phasic injection protocol (divided into an initial bolus of 40 mL at 4 mL/s followed by 20 mL of a 50%:50% mixture with NaCl at 4 mL/s), with bolus-triggering in the ascending aorta. For this purpose, a region of interest (ROI) was placed in the left atrium. Data acquisition began 7 seconds after the attenuation of the ROI reached 70 Hounsfield units (HUs) (a bolus tracking technique), with the retrospective ECG-gated data acquisition of the aortic root followed by a non-gated aortofemoral high-pitch spiral dual-source acquisition.

Postprocessing was completed at a designated workstation (Syngo Multimodalitiy Workplace, Siemens healthineers, Erlangen, Germany).

### 2.3. MRA Data Acquisition

All patients were examined using a 3T MRI system (Siemens Somatom Skyra, Siemens healthineers, Erlangen, Germany). Time-of-flight (TOF) MRA was used for the assessment of the femoral access route. The following scan parameters were applied: repetition time (TR) of 458 ms, echo time (TE) of 3.7 ms and flip angle of 50°. The slice thickness was 3.5 mm.

### 2.4. Image Review Methods

Two experienced readers (T.K., with 8 years of experience in both MRA and CTA, and P.R., with 3 years of experience in both MRA and CTA) independently evaluated the MRA measurements. They were blinded to the clinical findings, the medical histories of the patients and the CTA images. To avoid recall bias, the measurements for CTA were performed after a delay of two months; this was undertaken in consensus with the readers (T.K. and P.R.), who were blinded to the MRA measurements.

### 2.5. Assessment of the Access Route

**Figure 1:**
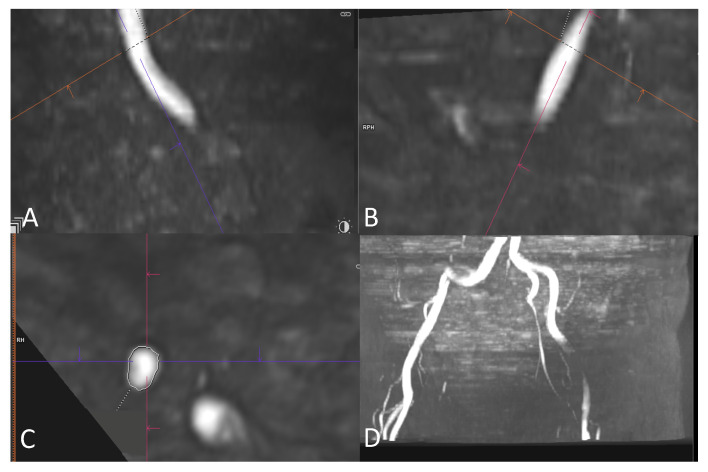
Exemplary MRA in multiplanar reformation showing angulation of two planes (**A**,**B**) at the site of the right external iliac artery to create a cross-sectional image perpendicular to the blood flow (**C**). Area was measured at the site of maximal lumen narrowing and minimal effective diameter was calculated as described above. (**D**) A 3D reconstruction of iliac vessels in MPR.

**Figure 2:**
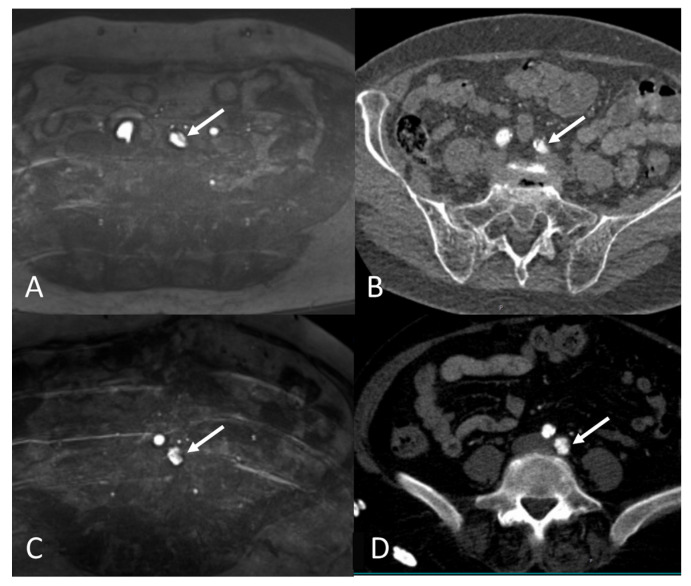
(**A**,**B**) MRA (**A**) and corresponding CTA (**B**) images of the same patient showing obstructing plaque (white arrow) in left common iliac artery preventing transfemoral access on left side. (**C**,**D**) MRA (**C**) and corresponding CTA (**D**) images of the same patient showing short dissection (white arrow) of left common iliac artery preventing transfemoral access on left side.

Our study protocol for MRA focused on the iliofemoral vessels; thus, it did not include an assessment of the aorta. All measurements were obtained from the common iliac artery (CIA), the external iliac artery (EIA) and the common femoral artery (CFA) of the iliofemoral vessels on each side. The minimal effective diameter and area were assessed for each segment. Multiplanar reformation (MPR) was used for the measurements of the minimal effective diameter. Two planes were used to create a cross-sectional image that was orthogonal to the blood flow direction. The area was measured on the cross-sectional image at the site of maximal lumen narrowing (Figure 1). The minimal effective diameter was calculated using the following formula: $minimal\;effective\;diameter=\left(\frac{\sqrt{Area}}{\pi }\right)*2.$ The feasibility of transfemoral access was evaluated according to the minimum effective vessel diameter (>5 mm), stenosis grading and kinking, as well as the existence of a dissection (Figure 2).

### 2.6. Image Quality Assessment of MRA

The subjective image quality of TOF-MRA was graded by both observers using a semi-quantitative five-point scale. A grade of 5 indicated an excellent image quality with a high signal without artifacts; 4 indicated a good-quality image with a high signal without artifacts or only minor artifacts; 3 indicated a moderate-quality image with a weak signal, but the exclusion of relevant stenosis was possible; 2 indicated a low-quality image with a low signal or the presence of artifacts and where stenosis could not be ruled out; and 1 indicated a non-diagnostic image.

### 2.7. Statistical Analysis

All statistical analyses were performed using R (version 4.0.4). The continuous variables were presented as the mean and standard deviation. Differences in measurements were evaluated using Bland–Altman plots (mean difference ± 1.96 SD) and tested using Student’s *t*-test or a Mann–Whitney U test, as appropriate. The MRA and CTA measurements were correlated using Spearman’s or Pearson’s rank tests, as appropriate. Interobserver variabilities of measurements were calculated using the intraclass correlation coefficient (ICC) with a two-way model. A comparison of the feasibility of transfemoral access using MRA and CTA was performed using an agreement and Cohen’s kappa. The irr package (version 0.84.1) was used to calculate the intraclass correlation coefficient and Cohen’s kappa. A *p*-value below 0.05 was considered to be statistically significant.

## 3. Results

### 3.1. Patient Characteristics

The MRA was performed after the CTA for TAVI evaluations of 51 patients with severe aortic stenosis (31 women (60.8%), mean age: 82.69 ± 5.69 years). The mean time interval between the CTA and MRA was 1.43 ± 2.39 days. In total, 60.7% of patients demonstrated impaired renal function (eGFR < 60 mL/min/1.73 m^2^). The mean eGFR was 54.2 ± 20.8 mL/min/1.73 m^2^. The baseline characteristics are presented in Table 1.

### 3.2. MRA Image Quality

The average scan time was 7.6 ± 1.6 min. The overall median subjective image quality of the MRA was 5 [IQR 4–5]; this was also 5 for the CTA [IQR 4–5]. In four patients, the image quality of the right transfemoral access route was inadequate for evaluation; three were due to artifacts in the CFA as a result of total endoprosthesis (TEP) of the hip. Similarly, when evaluating the left transfemoral access route, there were five patients with inadequate image quality; three were due to artifacts as a result of a hip TEP. For the evaluation of transfemoral access through the right CIA/EIA/CFA, the image quality (≥3) was adequate in 47/51 patients (92%); for the left CIA/EIA/CFA, the image quality was adequate in 46/51 (90%) patients. In 44/51 (86.3%) of patients, there was adequate image quality for a simultaneous evaluation of both the right and left sides (Table 2).

### 3.3. Effective Vessel Diameter

**Figure 3:**
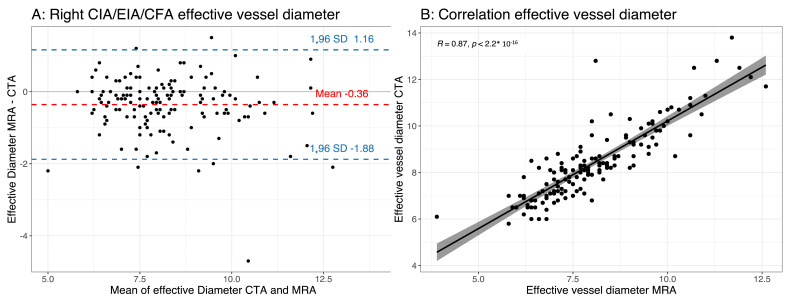
(**A**) Bland–Altman plot of effective minimal vessel diameter using magnetic resonance angiography (MRA) and computed tomography angiography (CTA) of right iliofemoral axis (common iliac artery, external iliac artery and common femoral artery (CIA/EIA/CFA), respectively); (**B**) scatter plot showing a good correlation between effective vessel diameter of right iliofemoral axis measured using MRA and CTA.

**Figure 4:**
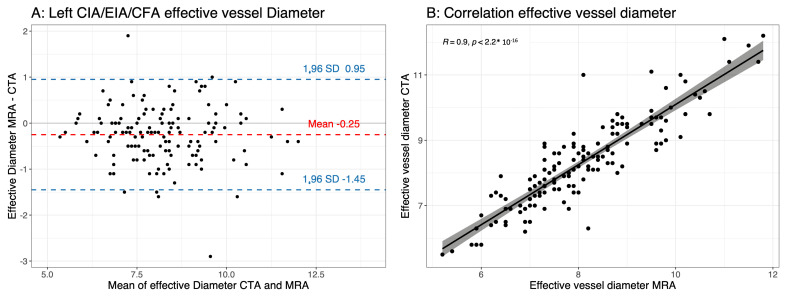
(**A**) Bland–Altman plot of minimal effective vessel diameter using magnetic resonance angiography (MRA) and computed tomography angiography (CTA) of left iliofemoral axis (common iliac artery, external iliac artery and common femoral artery (CIA/EIA/CFA), respectively); (**B**) scatter plot showing a good correlation between effective vessel diameter of left iliofemoral axis measured using MRA and CTA.

There was a significant difference in the measurements of the effective vessel diameter between the MRA and CTA. The MRA underestimated the effective vessel diameter compared with the CTA (the effective vessel diameter in mm for the right transfemoral access route (CIA/EIA/CFA) MRA vs. CTA was 8.04 ± 1.46 vs. 8.37 ± 1.54 (*p* < 0.0001) and the left transfemoral access route (CIA/EIA/CFA) MRA vs. CTA was 8.07 ± 1.32 vs. 8.28 ± 1.34 (*p* < 0.0001)) (Table 3, Figure 3 and Figure 4). The absolute difference between the MRA and CTA was small (Bland–Altman analysis of the right transfemoral access route (CIA/EIA/CFA) was −0.36 ± 0.77 mm (limits of agreement: −1.15; 1.87); Bland–Altman analysis of the left transfemoral access route (CIA/EIA/CFA) was −0.25 ± 0.61 mm (limits of agreement: 0.95; 1.44)). The overall correlation between the MRA and CTA measurements was very good (right transfemoral access route (CIA/EIA/CFA) Pearson correlation coefficient: 0.87 (*p* < 0.0001); left transfemoral access route (CIA/EIA/CFA) Pearson correlation coefficient: 0.9 (*p* < 0.0001)) (Table 3, Figure 3 and Figure 4). The same observation was noted when separately analyzing each vessel segment (CIA, EIA and CFA) for the left and right sides (Table 3). The interobserver agreement was excellent for the MRA measurements of the effective vessel diameter, with an ICC of 0.867 (95% confidence interval: 0.819–0.902) for the right side and an ICC of 0.929 (95% confidence interval: 0.902–0.948) for the left side (Table 4).

### 3.4. Feasibility of Transfemoral Access

The agreement in terms of the feasibility of transfemoral access was high between the MRA and CTA (Table 5). According to the MRA, transfemoral access was possible through the right access route in 46 out of 47 patients compared with 51 out of 51 patients when using the CTA (agreement of 97.9%, Kohen’s kappa was not applicable). Regarding the left access route, transfemoral access was possible in 44 out of 46 patients when using the MRA and 49 out of 51 patients when using the CTA (agreement of 95.7%, Kohen’s Kappa: 0.477 (*p* = 0.001)). There was one case of a short dissection of the left common iliac artery (Figure 2), which was diagnosed using both the MRA and CTA.

## 4. Discussion

The main findings of this study were as follows:

1. The non-contrast MRA protocol was reliable for an assessment of the femoral access route prior to the TAVI in comparison with the gold-standard CTA for most of the patients.
2. The non-contrast TOF-MRA underestimated the minimal vessel diameter compared with the CTA, but the absolute differences were small and did not affect the evaluation of the feasibility of the transfemoral access.

Recently, CTA has evolved as the gold standard for TAVI planning due to its capacity for the accurate visualization of aortic roots and access routes [5]. The application of an intravenous iodinated contrast agent is required for CTA, which could worsen kidney functions and lead to acute or chronic kidney failure. As kidney failure is a known risk factor for a worse outcome after TAVI [8,11], an imaging approach without a contrast agent could benefit patients with reduced kidney function, leading to better overall outcomes after TAVI. Hence, a contrast-free MRA evaluation of the aortic root and access route prior to TAVI could be an alternative for these patients. A contrast-free approach could also benefit patients with severe allergies to iodinated contrast agents. Our previous study demonstrated the feasibility of aortic root measurements using non-contrast MRA for prosthesis sizing [8]. An assessment of the access route was missing in this analysis.

### 4.1. Comparison with Different Non-Contrast MRA Techniques

Different sequences for non-contrast MRA are available. TOF-MRA is one of the oldest non-contrast MRA techniques; it remains a common and widely used sequence for evaluations of carotid arteries [12,13]. Newer techniques for the evaluation of peripheral artery disease, including quiescent-interval single-shot (QISS) MRA, have emerged; these benefit from shorter scan times and fewer image artifacts [12]. Data evaluating vascular access in TAVI populations using non-contrast MRA are sparse. Studies that evaluated the QISS-MRA approach before TAVI were conducted, but these studies included significantly fewer patients (26 patients and 5 patients with 10 healthy volunteers, respectively) [14,15]. To the best of our knowledge, our study had the largest patient cohort for the evaluation of vascular access anatomy before TAVI using non-contrast MRA. Our data revealed that TOF-MRA remains a viable option because the image quality was very good overall, with reasonable scan times. In 9 out of 102 access routes (9%), the image quality was inadequate for evaluation, mostly due to the artifacts of total hip prostheses (6/9 patients). Patients with a total hip prosthesis might not be suitable for a non-contrast MRA approach. This could be an important drawback to an MRA approach as a hip TEP is a common finding in elderly TAVI patients [16].

### 4.2. Feasibility of Transfemoral Access Using MRA and CTA

Based on the data from this study, the TOF-MRA underestimated the minimal vessel diameter compared with the CTA, but the absolute differences were small. This was in line with findings from a study that used QISS-MRA for the evaluation of transfemoral access routes, which revealed that the mean diameter of infrarenal aortas and iliofemoral vessels significantly differed [14], but the absolute differences were small and did not affect decisions regarding transfemoral access. One possible explanation for the consistent tendency toward a lower minimal vessel diameter when using MRA in our cohort in comparison with the CTA values might be the sensitivity of TOF-MRA to flow-related dephasing due to hemodynamically significant stenosis; this can lead to an overestimation of stenosis [12]. An underestimation of the minimal vessel diameter may be less problematic in a clinical setting because if transfemoral TAVI access is deemed feasible using MRA, CTA would yield the same results. If, in future studies, TOF-MRA revealed a diameter of slightly less than the required minimum of 5.5 mm for the feasibility of transfemoral access, an evaluation using CTA might also consider transfemoral access to be possible. Our data revealed slightly higher minimal diameters; this should be taken into account in borderline scenarios in future studies when using TOF-MRA before considering alternative, and possibly riskier, access routes, such as transapical or trans-subclavian approaches.

In addition to an evaluation of the access route, CTA also offers the possibility of detecting the features of higher periprocedural risks that could influence the procedural strategy or even prompt advisement against TAVI. Multiple studies demonstrated that there is an association between higher periprocedural risks and these imaging-derived factors [6]. Most of these features are related to the extent and location of calcification, especially within the left-ventricular outflow tract. This is related to the risk of an annular rupture, as well as paravalvular leakage and calcification of the aortic arch, which is related to the risk of a periprocedural stroke [6]. The visualization of calcification in MRI can be challenging; currently, there are no data for the prediction of periprocedural risks based on MRA. One study proposed the use of a native CT scan of the thorax in addition to non-contrast MRA to assess calcification and exclude any high-risk features [17].

### 4.3. Contrast-Induced Acute Kidney Injury

The risk of the development of an acute kidney injury after CTA prior to TAVI is higher for patients with pre-existing kidney disease and when higher volumes of an intravenous iodinated contrast agent are applied [18]. Recently, published data from the Society of Thoracic Surgeons/American College of Cardiology National Cardiovascular Transcatheter Valve Therapy Registry comprising data from 107,814 patients revealed that an acute kidney injury following TAVI remained a common finding and occurred in more than ten percent of patients [9]. Patients who developed a stage 3 acute kidney injury according to the acute kidney injury network criteria [19] had a seven-fold higher adjusted one-year mortality compared with patients who did not develop an acute kidney injury. Although the risk of a contrast-induced acute kidney injury following exposure to an intravenous iodinated contrast agent was overstated in the past for patients with normal or only mildly impaired renal functions, patients with severe kidney disease are considered to be at risk, as stated by consensus statements from the American College of Radiology and the National Kidney Foundation [20]. In patients with chronic kidney disease (CKD) stages 4 or 5 (eGFRs of 15–29 mL/min/1.73 m^2^ or 15 mL/min/1.73 m^2^, respectively), the potential risks and benefits, as well as alternative imaging strategies, should be carefully considered before performing contrast-enhanced computed tomography. The guidelines recommend volume expansion using normal saline as a prophylaxis in these patients to prevent an acute kidney injury prior to the administration of intravenous iodinated contrast media [20]. This recommendation cannot generally be applied to patients with severe aortic stenosis, as it could lead to the worsening of heart failure. Patients with chronic kidney disease stages 4 or 5 may benefit from a contrast-free imaging approach. Recently, there have been advances in performing the TAVI procedure itself without a contrast medium. A pilot study of 25 patients revealed that contrast-free transcatheter aortic valve implantation was feasible and safe [21]. These findings require confirmation using larger studies. A contrast-free TAVI procedure should be accompanied by a contrast-free imaging approach pre-TAVI. Non-contrast magnetic resonance angiography could be the ideal imaging modality to replace contrast-enhanced computed tomography in a zero-contrast approach for TAVI, as it permits the 3D visualization and reconstruction of the access route and has demonstrated promising results in recent studies.

### 4.4. Study Limitations

Although the patient cohort in this study was one of the largest on the subject of using MRA for the assessment of iliofemoral access routes prior to TAVI, the number of study participants was low in comparison with the data available from studies using CTA. The study protocol for MRA did not include an assessment of the aorta; contraindications on the level of the aorta (for example, severe kinking of the aorta) could not be evaluated using MRA.

Longer scan times when using MRA, especially TOF-MRA, might hinder its application for certain patients, especially older and more frail patients, where lying calmly in a supine position without moving for a long time might be difficult. Those patients might benefit from faster MRA sequences with shorter scan times, as provided by QISS-MRA.

The non-contrast MRA evaluation of transfemoral access is a promising approach for patients with chronic kidney disease who are referred for TAVI. Whether this translates into better outcomes for these patients is unclear, as there are no randomized data currently available on this subject. MRA is a more time-consuming and expensive technique compared with CTA; a substantial benefit to the patient would need to be demonstrated to justify its use. Further prospective, preferably randomized, studies are required before non-contrast MRA can be recommended for broader applications. In this context, data from an ongoing randomized trial are eagerly awaited [22].

## 5. Conclusions

The evaluation of iliofemoral access routes for TAVI using non-contrast MRA is feasible and reliable. Patients at risk of contrast-induced nephropathy might particularly benefit from such an approach. Whether this approach translates into better overall clinical outcomes after TAVI requires evaluation in future studies.

## Figures and Tables

**Table 1:** Baseline characteristics.

Age (years)	82.69 ± 5.69
Female, *n* (%)	31/51 (60.8%)
Body mass index (kg/m^2^)	27.0 ± 4.93
Body surface area (m^2^)	1.82 ± 0.22
Creatinine (mg/dL)	1.13 ± 0.52
eGFR (mL/min/1.73 m^2^)	54.2 ± 20.8
hs-cTnT (ng/L)	0.08 ± 0.18
Echo, LVEF (%)	52.5 ± 13.6
Contrast agent for CTA (mL)	58.5 ± 10.5
Atrial fibrillation, *n* (%)	22/51 (43.1%)

Data presented as mean ± standard deviation or as number and percentage of total. eGFR: estimated glomerular filtration rate; hs-cTnT: high-sensitivity cardiac troponin T; Echo, LVEF: left-ventricular ejection fraction assessed by echocardiography; CTA: computed tomography angiography.

**Table 2:** MRA parameters.

Average scan time (min)	7.6 ± 1.6
Median MRA image quality	5 (IQR 4–5)
MRA with sufficient image quality (≥3)	
Right CIA/EIA/CFA	47/51 (92%)
Left CIA/EIA/CFA	46/51 (90%)
Both sides simultaneously	44/51 (86.3%)

Data presented as mean ± standard deviation or median and interquartile range (IQR) of the magnetic resonance angiography (MRA) parameters. CIA: common iliac artery; EIA: external iliac artery; CFA: common femoral artery.

**Table 3:** MRA and CTA measurements of minimal effective vessel diameter.

	MRA	CTA	*p*-Value (*t*-Test)	Pearson Correlation Coefficient	*p*-Value (Pearson Correlation)	Bland–Altman Analysis
Diameter of right CIA/EIA/CFA	8.04 ± 1.46	8.37 ± 1.54	<0.0001	0.87	<0.0001	−0.36 ± 0.77
Diameter of left CIA/EIA/CFA	8.07 ± 1.32	8.28 ± 1.34	<0.0001	0.9	<0.0001	−0.25 ± 0.61
Diameter of right common iliac artery	9.08 ± 1.6	9.61 ± 1.68	<0.0001	0.86	<0.0001	−0.56 ± 0.88
Diameter of right external iliac artery	7.37 ± 1	7.68 ± 0.98	0.002	0.79	<0.0001	−0.3 ± 0.64
Diameter of right common femoral artery	7.64 ± 1.09	7.83 ± 1.04	0.047	0.75	<0.0001	−0.22 ± 0.75
Diameter of left common iliac artery	8.85 ± 1.27	9.07 ± 1.43	0.035	0.85	<0.0001	−0.23 ± 0.76
Diameter of left external iliac artery	7.57 ± 1.09	7.86 ± 1.08	0.0002	0.88	<0.0001	−0.31 ± 0.54
Diameter of left common femoral artery	7.75 ± 1.24	7.93 ± 1.16	0.0066	0.92	<0.0001	−0.2 ± 0.49

MRA: magnetic resonance angiography; CTA: computed tomography angiography; CIA: common iliac artery; EIA: external iliac artery, CFA: common femoral artery.

**Table 4:** Interobserver variability for MRA measurements.

	ICC (95% CI)
Right iliofemoral axis CIA/EIA/CFA minimal effective diameter	0.867 (0.819–0.902)
Left iliofemoral axis CIA/EIA/CFA minimal effective diameter	0.929 (0.902–0.948)

Interobserver variability by intraclass correlation coefficient (ICC) and lower and upper limits of the 95% confidence interval (CI) for interobserver variability of magnetic resonance angiography (MRA) measurements. CIA: common iliac artery; EIA: external iliac artery; CFA: common femoral artery.

**Table 5:** Feasibility of transfemoral access.

	MRA	CTA	Agreement	Kappa Value	*p*-Value
Right CIA/EIA/CFA	46/47	51/51	97.9%	-	-
Left CIA/EIA/CFA	44/46	49/51	95.7%	0.477	0.001

MRA: magnetic resonance angiography; CTA: computed tomography angiography; CIA: common iliac artery; EIA: external iliac artery; CFA: common femoral artery.

## Data Availability

The data are not publicly available due to containing information that could compromise the privacy of the research participants.
